# EasyModeller: A graphical interface to MODELLER

**DOI:** 10.1186/1756-0500-3-226

**Published:** 2010-08-16

**Authors:** Bhusan K Kuntal, Polamarasetty Aparoy, Pallu Reddanna

**Affiliations:** 1 School of Life Sciences, University of Hyderabad, Hyderabad 500 046, India

## Abstract

**Background:**

MODELLER is a program for automated protein Homology Modeling. It is one of the most widely used tool for homology or comparative modeling of protein three-dimensional structures, but most users find it a bit difficult to start with MODELLER as it is command line based and requires knowledge of basic Python scripting to use it efficiently.

**Findings:**

The study was designed with an aim to develop of "EasyModeller" tool as a frontend graphical interface to MODELLER using Perl/Tk, which can be used as a standalone tool in windows platform with MODELLER and Python preinstalled. It helps inexperienced users to perform modeling, assessment, visualization, and optimization of protein models in a simple and straightforward way.

**Conclusion:**

EasyModeller provides a graphical straight forward interface and functions as a stand-alone tool which can be used in a standard personal computer with Microsoft Windows as the operating system.

## Findings

Structural information of biological macromolecules is readily available in the Protein Data Bank (PDB) [1], http://www.pdb.org. By Sep 2009, the PDB contained ~ 60,713 experimental protein structures that can be grouped into ~ 3500 families [2]. Considering that the number of non redundant amino acid sequence entries is around 408,000 http://www.expasy.org/sprot/, there is a huge gap between known annotated sequences and available 3 D structures. Developments in genomics have also spurred the developments in X-ray crystallography and NMR techniques to solve the new protein structure, which in turn has widened their use in drug discovery [3]. However, these efforts are no where near in solving the 3 D structures of all the known proteins in any system. In the absence of experimental structures, computational methods are used to predict 3 D protein models to provide insight into the structure and function of these proteins. The steps involved in this process are [4]: (1) identification of homolog that can be used as template(s) for modeling; (2) alignment of the target sequence to the template(s); (3) backbone generation; (4) loop modeling; (5) side-chain modeling; (6) model optimization; and (7) validation of the model. Repositories like The SWISS-MODEL http://swissmodel.expasy.org/SWISS-MODEL.html, Protein Model Portal http://proteinmodelportal.org[5] and Modbase http://modbase.compbio.ucsf.edu[6], contain protein models generated using various auto-mated methods. However, without human intervention, errors as a result of inaccurate sequence alignment, and inability to identify and correctly model domains, such as loop and ligand-binding regions, are magnified, which results in the generation of low-accuracy models and thus limiting their applicability to drug discovery projects [7,8]. In this context, the development of various user friendly and accurate tools for homology modeling is an active area of research such as new recent tools like HHPRED and Modeller at http://toolkit.tuebingen.mpg.de/sections/tertstruct toolkit, GeneSilico https://genesilico.pl/toolkit/[9-15]. MODELLER is one of the most widely used tools for homology or comparative modeling of protein three-dimensional structures. MODELLER stands apart from other packages due to its free availability, powerful features and reliable results. But most users find a bit difficult to start with MODELLER as it is command line based. Hence a freely available GUI for MODELLER would thus be very helpful to exploit the powers and advantages of this package more effectively. EasyModeller is a graphical user interface to MODELLER program.

**Figure 1:**
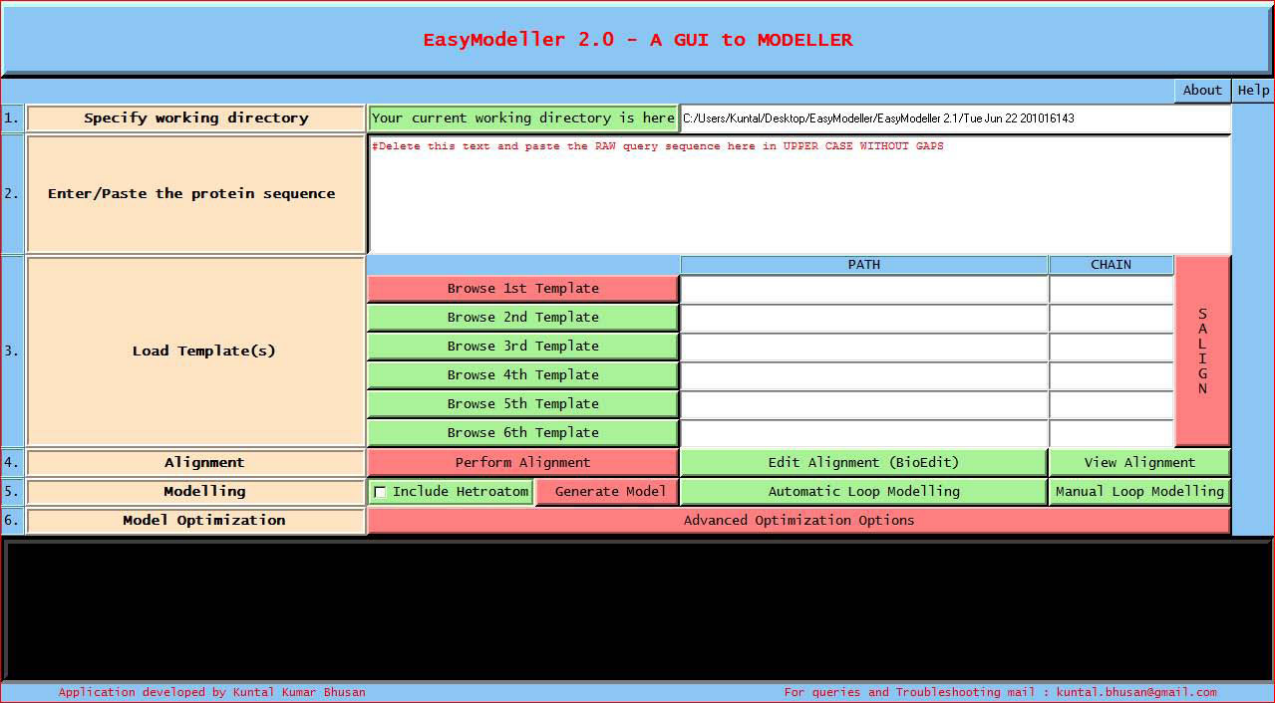
**Screenshot of the tool**.

EasyModeller is a standalone tool with a very intuitive interface which clearly defines the different steps of homology modeling (Additional file 1). The screenshot of the tool is shown in Fig. 1 which shows six steps required for building a homology model with the help of EasyModeller. User is required to follow the numbered steps one by one, which is guided by associated help information. A blue clickable panel called the "Help panel" can be used to view the help tips associated with each step. EasyModeller follows a very simple color coding consisting of green and red buttons. The features (buttons) marked red are the minimum compulsory steps to get a model while those in green are the optional ones.

**Figure 2:**
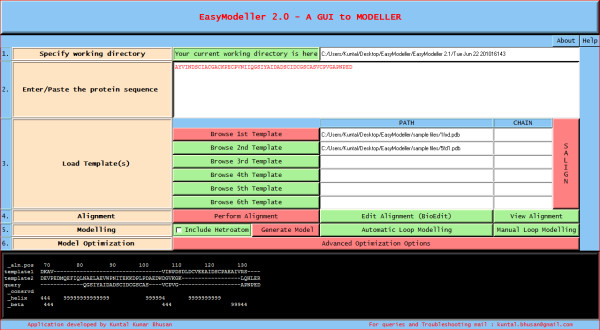
**Multi template modeling**.

**Figure 3:**
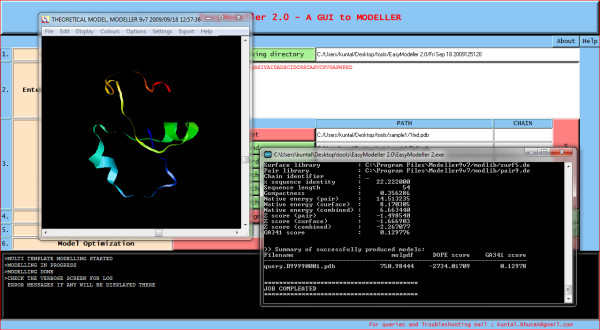
**The best model built shown in the default PDB viewer**.

**Figure 4:**
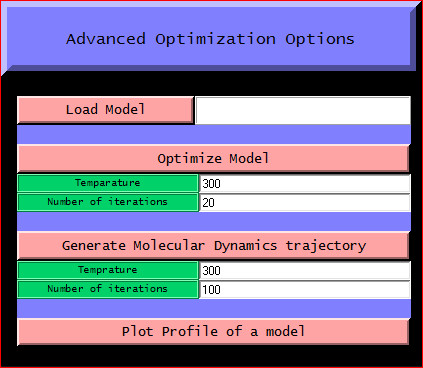
**Advanced optimization options**.

**Figure 5:**
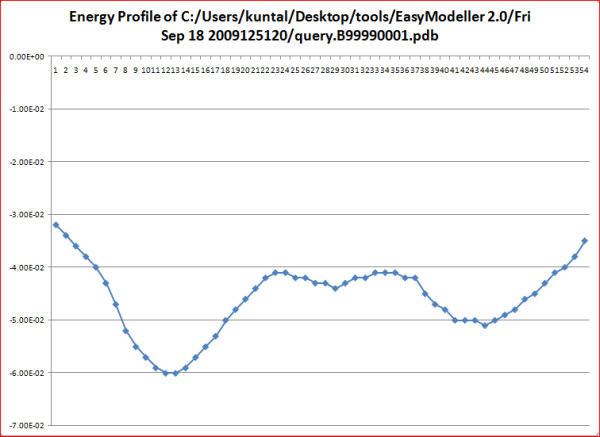
**Energy profile graph**.

The first step involves specifying the working directory, which is the folder location where the output files will be generated. This working directory will also help to keep a track of all the generated files. The second and most basic step is entering the amino acid sequence information as the input parameter. The third step is providing the template information to the program. The user can load the template structure(s) in standard formats like (.pdb, .ent, etc) acceptable in MODELLER by using the Load template(s) feature. The basic template information like its name, chains, heteroatoms, etc are shown in the display area and the CHAIN is automatically set default to the first chain as set in the PDB file. To use a different chain for the template the text box containing the chain information can be edited and the desired chain id can be entered. The text box is automatically kept blank if no chain information is found. To do multi template based modeling users can load all the template structure files one by one in order with a maximum of six templates as in Fig 2. The next step of homology modeling is aligning the query sequence with the template which is achieved in step four. The "Perform Alignment" feature aligns the query sequence with the template(s) using the align2 d function of MODELLER and displays the output alignment in the text display window of the tool. Although the display area is large enough, sometimes the output display might seem not to fit in it. So the display is made scrollable, users just need to double click on it to activate the feature and scroll the mouse wheel down to view the entire display contents. A beneficial feature of the tool is the possibility to view and manually improve the query alignment via the feature 'Edit Alignment'. Although the tool provides a preliminary option for alignment editing, for users who would like to use advanced visual alignment editing can install BioEdit [16] and manually open the appropriate alignment (.ali) files from the current working directory with BioEdit (by one time associating .ali files with the tool during installation), do the editing on the go and save them in the same location. The fifth step is generating the homology model by using the information generated so far. The "Generate Model" feature is used to achieve this by using the appropriate MODELLER function as required. As soon as the model is generated, the best model is displayed in the users default PDB viewer like Rasmol [17] as in Fig. 3. Further the generated model can be improved upon by loop modeling. MODELLER has several loop optimization methods, which all rely on scoring functions and optimization protocols adapted for loop modeling [10]. The sixth and the final step is model optimization which can be achieved by using the advanced optimization options Fig 4. The various parameters for optimization and dynamics like temperature and number of iterations can be changed by editing the default value in the corresponding text boxes. The minimized models are generated and are saved in the working directory inside a new folder called optimized models. The dynamics output binary trajectory files are also saved in the same folder which can be read in by visualization software such as CHIMERA [18] or VMD [19]. Further the model profile plot can be generated by selecting the "Plot profile of a model" option which calculates DOPE energy of the loaded model using the assess_dope function [20] and displays it as in Fig. 5.

The GUI eliminates the requirement of prior knowledge in the backend applications, thereby increasing the number of users of MODELLER and assists them to exploit the unique features of this great package more effectively. EasyModeller uses default parameters for most commands during software execution to make the process as simple as possible. User can change the parameters manually by editing the associated python script file (*.py) generated in the working directory.

EasyModeller will be updated by adding features like comparison and manual combination of multiple template structure and manual definition of spatial restraints into a more powerful GUI to MODELLER which could simultaneously display both alignment and structure windows, and have them interact with each other.

## Availability and requirements

**Project name: **EasyModeller

**Project homepage: **http://www.uohyd.ernet.in/modellergui/

**Operating system: **Microsoft Windows (any)

**Programming language: **Perl (using Perl/Tk)

**Other requirements: **The system must have MODELLER (any version will work but preferably the latest version to get the best results) and Python (2.5 or 2.6 and not 3.11) preinstalled in the default installation directory (C://Program files/..). If the operating system is Windows Vista or Windows 7 then please run the executable file (.exe) of EasyModeller with administrative privilege (by right clicking it and selecting "Run as administrator"). Since EasyModeller uses the Microsoft Excel plot function to plot the profile graph, it is necessary to have Microsoft Excel installed in the system. A PDB viewer like Rasmol is required to visualize the generated model.

**License: **Free to use

**Any restrictions to use by non-academics: **None

## Competing interests

The authors declare that they have no competing interests.

## Authors' contributions

BKK carried out the planning and development of the GUI, PA contributed in the enhancement of GUI and in manuscript preparation and PR coordinated the whole work.

## Supplementary Material

Additional file 1 **This file contains the EasyModeller tool executable file and the details of the tool**. Click here for file
